# A bending rigidity parameter for stress granule condensates

**DOI:** 10.1126/sciadv.adg0432

**Published:** 2023-05-17

**Authors:** Jack O. Law, Carl M. Jones, Thomas Stevenson, Thomas A. Williamson, Matthew S. Turner, Halim Kusumaatmaja, Sushma N. Grellscheid

**Affiliations:** ^1^ Computational Biology Unit and Department of Biological Sciences, University of Bergen, Bergen, Norway.; ^2^ Department of Biosciences, University of Durham, Durham, UK.; ^3^ Department of Physics, University of Durham, Durham, UK.; ^4^ Department of Physics, University of Warwick, Coventry, UK.

## Abstract

Interfacial tension plays an important role in governing the dynamics of droplet coalescence and determining how condensates interact with and deform lipid membranes and biological filaments. We demonstrate that an interfacial tension-only model is inadequate for describing stress granules in live cells. Harnessing a high-throughput flicker spectroscopy pipeline to analyze the shape fluctuations of tens of thousands of stress granules, we find that the measured fluctuation spectra require an additional contribution, which we attribute to elastic bending deformation. We also show that stress granules have an irregular, nonspherical base shape. These results suggest that stress granules are viscoelastic droplets with a structured interface, rather than simple Newtonian liquids. Furthermore, we observe that the measured interfacial tensions and bending rigidities span a range of several orders of magnitude. Hence, different types of stress granules (and more generally, other biomolecular condensates) can only be differentiated via large-scale surveys.

## Teaser

High-throughput analysis of interface fluctuations of stress granules reveals a bending rigidity parameter.

## INTRODUCTION

Biomolecular condensates are membraneless subcellular compartments enriched in specific types of biomolecules. The eukaryotic cell has nuclear condensates such as nucleoli and speckles with roles in regulating nucleic acid metabolism (*1*–*3*). In the cytoplasm, condensates are frequently formed in response to cellular stress or altered RNA homeostasis such as in the case of stress granules, p-bodies, and signalosomes (*4*, *5*). In addition, dysregulation of condensates leading to a liquid-to-solid phase transition due to the formation of fibrils has been implicated in numerous neurodegenerative diseases such as amyotrophic lateral sclerosis (*6*). It is now widely recognized that condensates form through a process of macromolecular liquid-liquid phase separation (LLPS) of cellular components, primarily proteins and nucleic acids (*7*–*9*). Moreover, biomolecules that are multivalent can drive a networking transition called percolation or gelation (*6*). LLPS and percolation act synergistically to give rise to these subcellular bodies that are variously referred to as granules, membraneless organelles, biomolecular condensates, or simply condensates.

Condensates are thought to act as reaction crucibles and sites of chemical sequestration (*7*). Their ability to carry out these functions is closely related to their mechanical properties, both at the interface, such as interfacial tension (sometimes referred to as surface tension, although strictly surface tension only refers to gas-liquid surfaces), and in the bulk, such as viscosity. These strongly influence the rates of biochemical reactions as well as the coalescence and growth of condensates. More recently, there has also been a growing recognition that condensates interact with, deform, and remodel one another, as well as other cellular components, including lipid membranes and cytoskeletal filaments (*2*, *10*–*17*), among others. This further brings to the fore the importance of interfacial properties in determining condensate shape changes.

Our work here aims to address two major open challenges in the field of condensates. First, recent works have provided some evidence that condensates are not simple Newtonian liquid droplets, suggesting that they have a complex rheological behavior (*8*, *18*) and that they may have a structured interface (*19*–*21*). In the context of condensate shape deformation and its interaction with other cellular components, this raises the important question of whether there are relevant mechanical forces at the interface beyond interfacial tension.

Second, despite the widespread importance of interfacial tension for the interactions between biomolecular condensates and other cellular components, current techniques for their measurement in live cells have a number of limitations. Standard interfacial tension methods such as sessile drop tensiometry, Du Noüy ring, or Wilhelmy plate (*22*–*24*), while highly accurate for measurements in vitro, are not applicable in living cells. Pioneering studies in vivo have estimated the interfacial tension of condensates in living cells from the time scale of droplet coalescence events (*2*). Typically, only a handful of events are analyzed, because identifying them in live-cell imaging is cumbersome. Furthermore, this approach uses an assumption that such a time scale depends on a simple viscosity-interfacial tension ratio (*1*, *25*–*27*), and it requires the condensate viscosity to be known. Measurements of condensate viscosity are often based on fluorescence recovery after photobleaching, which itself is known to have a number of drawbacks, including a strong dependence on the area bleached and choice of fluorescence recovery model (*28*–*32*). Although these methods have proved useful to provide an order of magnitude estimate for the interfacial tensions of condensates, the emergence of alternative approaches would open up additional possibilities in condensate research.

To address both of these challenges, in this work, we have developed a high-throughput approach based on flicker spectroscopy to study stress granules as a model condensate. Stress granules are cytoplasmic condensates formed as a response to cellular stresses such as heat shock or the presence of toxins. Their role is to arrest translation, which is achieved by sequestering mRNA (*33*, *34*). Furthermore, misregulation of stress granules is associated with neurodegenerative diseases (*35*). While we have used stress granules as a model condensate, the presented approach would be widely applicable to any liquid-like condensate in living cells.

The basis of flicker spectroscopy is the analysis of the fluctuations in the shape outline of liquid-like condensates from high-resolution live-cell microscopy. The application of flicker spectroscopy is well established for several systems such as for colloid-polymer mixtures (*36*) and lipid bilayers and vesicles (*37*). For condensates, previously, Caragine *et al*. (*25*) have used the average interface fluctuations to estimate the interfacial tension of nucleolar condensate. Here, we expand on this approach for condensates in two important ways. First, we analyze the condensate fluctuation spectra rather than just using the average fluctuation amplitude, which means that we can investigate whether there is any other relevant mechanical force other than interfacial tension. We highlight the role of elastic bending deformation in the mechanics of stress granules, which supports the view that condensates are not simple liquid droplets. This is characterized by the bending rigidity, which is the energy penalty required for deforming the condensate interface. Second, we establish a high-throughput protocol that provides a quantitative analysis on the values of interfacial tension and the bending rigidity term over tens of thousands of individual stress granules. This is a step change from previous studies that could only study a small number (~10 s) of individual condensates (*1*, *25*, *27*). We find that both the interfacial tension and bending rigidity have wide distributions, but they can be well characterized. Our large-scale surveys allow us to distinguish stress granules induced by different chemicals or under different stoichiometries of constituent proteins based on measured distributions of the material properties.

## RESULTS

### Automated image analysis

**Fig. 1.**
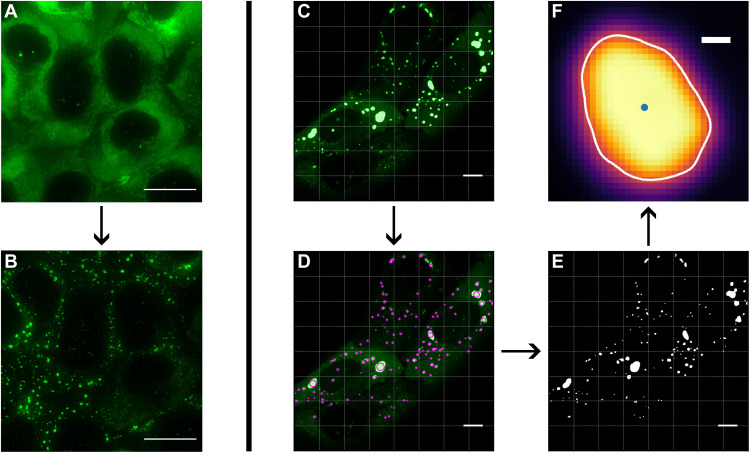
The formation and detection of stress granules. U2OS cells endogenously expressing G3BP1-GFP (**A**) form stress granules upon treatment with 200 μM sodium arsenite from 15 to 20 min onwards (**B**). Scale bars, 20 μm (A and B). Stress granules are imaged over a period of up to 2 hours, with each region imaged for 40 s; collecting 1000 frames. The resulting videos, illustrated by a frame in (**C**), are analyzed frame by frame to detect granules and identify their boundaries. The difference-of-Gaussians algorithm identifies the centers of the granules in the image and their approximate size. The granules are then tracked from frame to frame to record their fluctuations over time (**D**). A flood fill is used to estimate the extent of each granule (**E**). The boundary of each granule for each frame is identified by finding the maximum of the directional gradient along lines radiating from the center of the granule. Scale bars, 10 μm (C to E). An example of a fully analyzed granule is shown in (**F**). Scale bar, 0.5 μm (F).

We image U2OS cells stably expressing Ras GTPase-activating protein-binding protein (G3BP1) tagged with Green Fluorescence Protein (GFP) and lacking endogenous G3BP1 and G3BP2 (*38*). G3BP is a protein that is spread throughout the cytoplasm under normal circumstances, but localizes to the stress granules when they are formed, and can therefore be used to visualize them. In this section, stress granules are induced using 200 μM sodium arsenite. Figure 1 (A and B) shows typical examples of the cells before and after the introduction of sodium arsenite. They are then imaged using a spinning disk microscope. We observed that, because of phototoxicity, if a single region is imaged for too long, then the laser itself begins to trigger stress granule formation. Therefore, we image each region for only 40 s. See Materials and Methods for cell culture and imaging details. We apply a bespoke image analysis algorithm to these videos to extract the outline of each granule across the frames. This analysis yields *D*(φ), the distance from the center of the granule to the outline as a function of polar angle φ around the center. This procedure is illustrated in Fig. 1 (C to F) and described in Materials and Methods.

### Fitting experimental and theoretical spectra: The emergence of bending rigidity as a material parameter

**Fig. 2.**
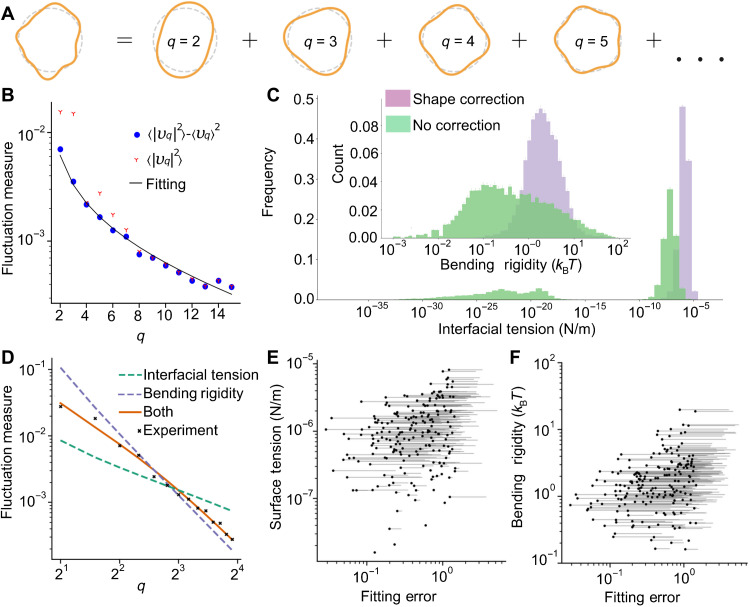
Fitting the fluctuation spectrum. (**A**) The projected outline of a granule is decomposed into its Fourier modes for each frame. The amplitude of each mode is combined across the frames to give the average ⟨∣*υ_q_*∣^2^⟩ (total surface fluctuations) and the variance ⟨∣*υ_q_*∣^2^⟩ − ∣⟨*υ_q_*⟩∣^2^ (base shape–corrected fluctuations). (**B**) The measured fluctuation spectrum of an example stress granule (red Ys), the spectrum after correction for the nonspherical base shape (blue dots), and the fitted model spectrum (black line). The fitting method is described in section SM2 in the Supplementary Materials. (**C**) The effect of the shape correction across a population of 12,163 stress granules (see next section for details of granule selection). It can be seen that correcting for the shape both reduces the width of the histogram and removes a large population of granules erroneously identified with a very low interfacial tension. Here, we measure the bending rigidity in the unit of *k*_B_*T*, where *k*_B_ is the Boltzmann constant and T is the temperature. (**D**) Another example of a fluctuation spectrum (black crosses) with fitting to three theoretical spectra, accounting for contributions from interfacial tension only (green dashes), bending rigidity only (purple dash), and both interfacial tension and bending rigidity (orange line). Only the model with both interfacial tension and bending rigidity fits well to the experimental data. (**E**) The change in fitting error when the bending rigidity is removed. For clarity, we select 300 granules at random. The solid point is the fitting error with bending rigidity. The thin line is drawn from that point to the error to the fit when bending rigidity is neglected. (**F**) Similar analysis but for when the interfacial tension is removed. Both in (E) and (F), the fitting is markedly worse without interfacial tension or bending rigidity.

The shape deformation of a stress granule boundary can be decomposed into its independent Fourier modes (*q*) as shown in Fig. 2A. A Fourier transform of *D*(φ) gives the fluctuation spectrum with *υ_q_* the magnitude of the *q*th fluctuation mode and *q* a positive integer. Because of the resolution of the microscope, only the first 15 modes can be reliably resolved for the smallest stress granules, so we truncate the spectrum at *q* = 15 (see Materials and Methods).

To infer the condensate interfacial tension and bending rigidity, the measured fluctuation spectra must then be fitted to a theoretical prediction. The choice for the fitting error function is discussed in section SM2 in the Supplementary Materials. Here, we find that they fit well to a spectrum derived from the Helfrich-free energy (*39*)

$$HE=\int_{a}dA\left[\frac{\kappa }{2}H^{2}+\sigma \right]$$ (1)

where κ is the bending rigidity, *H* is the total curvature, σ is the interfacial tension, and the integral is over the area of the granule. The Helfrich-free energy is a widely used quadratic order approximation of the energy of an interface, valid so long as the curvature of the interface is gentle relative to the size of the interfacial molecules. This formulation assumes that the granules have no spontaneous curvature and no saddle-splay modulus. For a simple liquid droplet, we would expect no contribution from bending rigidity. However, a number of works have recently suggested that condensates are not simple liquids. Computational investigations have shown that even single-component droplets can have an inhomogeneous, viscoelastic interior and a structured interface (*40*, *41*). Experimentally, recent works have also reported intrinsically disordered proteins acting as pickering stabilizers on P granules (*19*) and farnesoid X receptor 1 (FXR1) proteins affecting the stoichiometry at the droplet interface (*20*, *21*).

When projected to the two-dimensional (2D) imaging plane, as is the case in our setup, the time-average fluctuation spectra are given by

$$\langle {|\nu_{q}|}^{2}\rangle =\frac{k_{B}T}{\kappa }\sum_{l=q}^{l_{\max}}\frac{N_{lq}^{2}P_{lq}^{2}(0)}{\left(l+2)(l-1)[l(l+1)+\bar{\sigma }\right]}$$ (2)

where *υ_q_* is the magnitude of the *q*th fluctuation mode, *k*_B_ is the Boltzmann constant, *T* is the temperature, *P_lq_* and *N_lq_* are the associated Legendre polynomials and normalization factor, respectively, and $\bar{\sigma }=\sigma R^{2}/\kappa$ is the dimensionless interfacial tension, with *R* the condensate radius (see section SM1 in the Supplementary Materials for a detailed derivation) (*37*). Typically, *l*_max_ = 75 is sufficient for the sum to converge.

A comparison of the measured and predicted spectra of the stress granules above led to the following insights. First, we find that the base shape of the stress granules is not perfectly spherical, at least over the duration of the measurement. This issue can be detected by showing that the average ⟨*υ_q_*⟩ is nonzero. To rectify this issue, we follow the approach by Pécréaux *et al*. (*37*) and expand our description of each fluctuation mode to include a fluctuating term *F_q_* and a constant term *C_q_*, such that we can write

$$\upsilon_{q}(t)=F_{q}\cos(\omega_{q}t+\delta_{q})+C_{q}$$ (3)

The fluctuating and constant components of the perturbations can be extracted from the measured fluctuations with

$$\begin{array}{c} {|F_{q}|}^{2}=\langle {|\upsilon_{q}|}^{2}\rangle -{|\langle \upsilon_{q}\rangle |}^{2}\\ {|C_{q}|}^{2}={|\langle \upsilon_{q}\rangle |}^{2} \end{array}$$ (4)

∣*F_q_*∣^2^ can then be used in place of ⟨∣*υ_q_*∣^2^⟩ as the fluctuation measure for calculating σ and κ. ∣*C*_2_∣^2^ gives an estimate of how elongated a granule is and can be used as a circularity measure. Failing to account for this base shape correction from a perfectly spherical liquid droplet to a spheroid leads to a spectrum that fits poorly to the theoretical model, especially for the lower fluctuation modes, as shown in Fig. 2B. Figure 2C shows the effect of this correction across a population of sodium arsenite induced stress granules. Ignoring the shape correction described in Eq. 4 leads to large misestimation of the interfacial tension and bending rigidity, including falsely identifying many droplets with negligible interfacial tensions. This finding provides support to the argument that stress granules are not simple liquids, where the average shape is expected to be spherical.

Second, if stress granules are simple liquid droplets, then their fluctuations are expected to depend mainly on interfacial tension. However, as shown in Fig. 2D, we observe that the resulting fit from including interfacial tension alone (setting κ = 0) is very poor. This is especially so for higher order *q* modes, where the bending term is expected to dominate (see Eq. 2). Similarly, the measured and predicted spectra are in poor agreement if only elastic bending deformation is taken into account (setting σ *= 0*), especially for the lower *q* modes. Figure 2 (E and F) quantifies the increase in fitting errors when either interfacial tension or bending rigidity is ignored for 300 independent measurements of sodium arsenite–induced stress granules. We find that bending rigidity is an essential parameter for describing the mechanical properties of stress granules and is likely relevant for any other biomolecular condensate.

Note that, for around 4% of granules, the inclusion of bending rigidity does not improve the fit at all. As seen in Fig. 2D, bending rigidity primarily affects the spectrum fit at large *q* modes with a crossover point that scales as $\bar{\sigma }=\sigma R^{2}/\kappa$. For a small minority of granules, the crossover point is beyond the *q*_max_ = 15 cutoff set by the resolution of our confocal microscopy. Therefore, we are unable to access the high *q* modes where elastic bending deformation dominates. Because the estimated bending rigidity for these granules is essentially arbitrary, we exclude these granules from our analysis (quantified in the next section below).

**Fig. 3.**
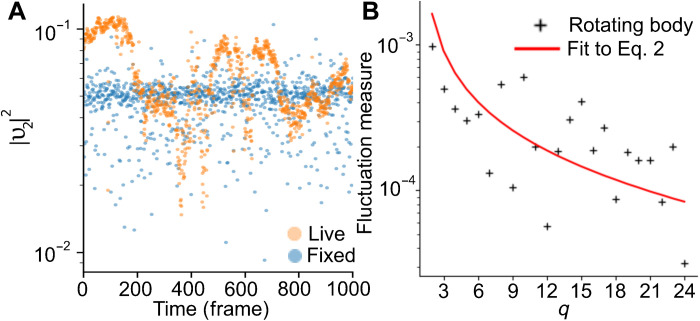
Validating the fluctuation spectrum. (**A**) In a live cell, the condensate boundary shows correlated fluctuations over time (orange), whereas in a fixed cell, only microscope noise is seen (blue). Each time between frames is approximately 0.04 s. (**B**) In a simulated, nonfluctuating granule undergoing random rotations, the theoretical model does not fit to the simulated spectrum.

We further find that the spectra observed from live cells are distinct from those obtained from identical fixed cells, demonstrating that the observed fluctuations are not just due to microscope noise, as illustrated in Fig. 3A, for *q* = 2. In addition, we eliminated the possibility that the fluctuations that we see are caused by rotations of a fixed shape droplet. To do this, we simulated randomly rotating rigid bodies with quenched shape roughness (see Materials and Methods for details). As shown in Fig. 3B, this has a different spectrum and fits poorly when compared to the model discussed above, indicating that the spectra measured from live cells are meaningful.

### High-throughput stress granule analyses: Dependencies on size, shape, and time

**Fig. 4.**
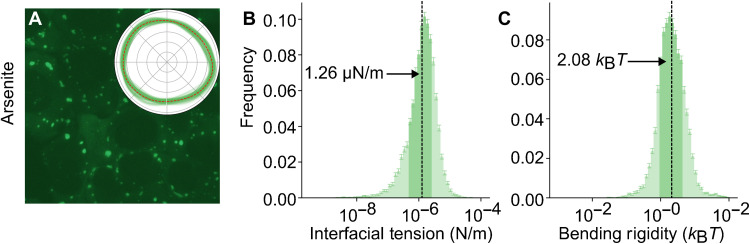
Experimental results for the high-throughput analysis of sodium arsenite–induced stress granules. (**A**) An overview image of sodium arsenite granules taken a few minutes after treatment. The inset shows the variation of the boundary of a representative granule over the course of the experiment, sampled every 10 ms. The red dashed line shows the average position of the boundary. (**B**) A histogram of the measured interfacial tensions of 12,163 stress granules that pass the filtering. The geometric mean is indicated with an arrow. The darker area represents the 67% of granules closest to the geometric mean. (**C**) A similar histogram for the bending rigidity of the stress granules.

Harnessing the automated granule detection and fluctuation analysis described above presents us with an opportunity to analyze the shape, size, interfacial tension, and bending rigidity of large populations of stress granules. We collect microscopy videos of 178,949 sodium arsenite–induced granules over two independent experimental runs (see Fig. 4A as an example). Figure S4 shows the 2D histogram of stress granules captured by confocal microscopy as a function of their size (mean radius) and shape (∣*C*_2_∣^2^, an estimate of the circularity; see Eq. 3 for the definition of *C*_2_). After shape fitting their boundaries, we filter out all granules lacking a closed outline in at least 60% of the frames. These 44,524 granules are further filtered to exclude those whose fitting is not affected by bending rigidity, meaning that the difference between the fitting errors (given by eq. S15 in section SM2 in the Supplementary Materials) with and without bending rigidity is less than 0.03. This leaves 42,894 granules. Last, we remove all granules where the error on the fit to the theoretical spectrum was greater than 0.5 (see section SM2 in the Supplementary Materials), typically corresponding to large, irregular shaped granules, resulting in a final selection of 12,163 stress granules. The size and circularity distribution for the prefiltered granules is shown in fig. S4.

We find that the measured interfacial tension spans across several orders of magnitude, with an approximately log normal distribution (see Fig. 4B). Taking the geometric average of the interfacial tension, we find an average value of 1.26 μN/m, with 67% of the data (equivalent to 1 SD in the log normal distribution) falling between 0.530 and 3.08 μN/m. These values are comparable to estimates in the literature for the nucleolus and P granules (*2*, *25*, *26*), which are around 1 μN/m. These values are much lower compared to the surface tension of common liquids (e.g., water-air surface tension is 72 mN/m). A possible explanation is that, in thermal systems, the interfacial tension value is expected to be approximately inversely proportional to the area of the macromolecular building blocks and proteins and RNAs are much larger than water molecules (*42*, *43*). A similarly low value of interfacial tension has also been reported for colloid-polymer mixtures for the same reason (*36*). Carrying out similar analysis for the bending rigidity (see Fig. 4C), 67% of the data range between 0.934 and 4.80 *k*_B_*T*, with a geometric average of 2.08 *k*_B_*T*, which is consistent with thermodynamically stable aggregates at the interface (*44*).

**Fig. 5.**
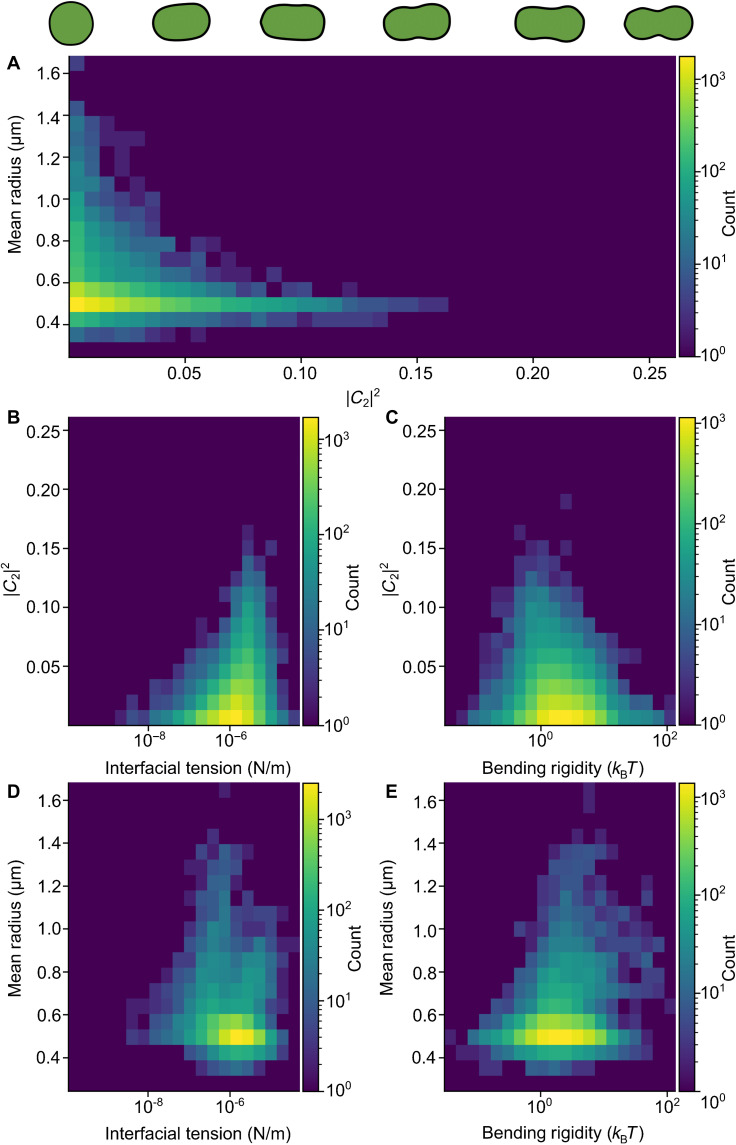
The relationships between sodium arsenite granule properties. (**A**) Distribution of granules as a function of their mean radius and circularity measure (∣*C*_2_∣^2^). Smaller droplets have a high range of circularities than larger droplets. This is because filtering effectively removes large irregular drops. We postulate that these represent mature, solid-like granules. The illustrated shapes correspond to different values of ∣*C*_2_∣^2^. (**B**) Less circular droplets have higher interfacial tension, and rounder droplets have a wider range of interfacial tensions. (**C**) Bending rigidity does not appear to be correlated with roundness, but rounder granules have a wider range of interfacial tensions. (**D**) There is no strong correlation between interfacial tension and mean radius. The spread of interfacial tension values is wider at lower radii. (**E**) Likewise, there is a wide spread of bending rigidity at low radius but a narrower range at higher radii. In both panels (D) and (E), measurements are concentrated at the lower radii.

Figure 5A shows the 2D histogram of size versus circularity of stress granules that pass the above filters and are successfully analyzed using flicker spectroscopy. We observe that small spherical stress granules are the most abundant, although stress granules ranged from 0.4 to 1.6 μm in size. Comparing Fig. 5A and fig. S4, it is clear that the flicker spectroscopy approach can be reliably applied to measure the interfacial tension and bending rigidity of spheroid stress granules, which are expected to display liquid-like behavior, whereas in contrast, large irregular shaped stress granules fail to pass the filters. Our observations are consistent with a number of works that have reported that, as stress granules mature, they assume a more irregular shape and potentially solid-like behavior with little to no fluctuations (*18*, *45*), which makes them unsuitable for shape fluctuation analysis. As we have highlighted in Fig. 3B, randomly rotating rough objects have a different spectrum to fluctuating liquid-like stress granules in live cells. Mature stress granules are also typically larger, as they are likely to have undergone merging events and/or have more time to grow in size. It is precisely these large, irregular granules that are removed by our filtering steps.

Next, we investigate the distributions of the measured interfacial tension and bending rigidities as a function of the granules’ radius and circularity. First, as shown in Fig. 5 (B and C), spherical granules have a larger range of interfacial tension and bending rigidity values. With decreasing circularity (larger ∣*C*_2_∣^2^), these ranges of values become more narrow. There is no clear correlation between circularity and the average value of bending rigidity, but we can observe a weak but noticeable tendency that less spherical granules have, on average, a higher interfacial tension. Second, analyzing the effect of size, shown in Fig. 5 (D and E), a similar narrowing down of the range is observed with increasing granule mean radius, but there is no clear correlation for the means of the interfacial tension and bending rigidity. We find that the qualitative behavior described above considering all data was unchanged for a subset of data analyzed as a function of granule size for a given circularity value.

**Fig. 6.**
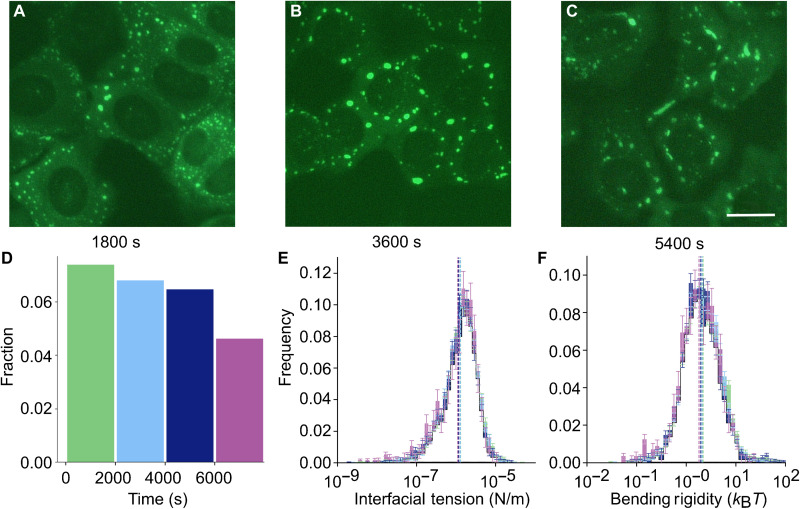
Changes in granule properties over time. (**A** to **C**) Example images of sodium arsenite–induced stress granules at *t* = 1800, 3600, and 5400 s after the introduction of the sodium arsenite. Scale bar, 25 μm. (**D**) Fractions of stress granules that pass filtering in four time domains: 0 to 2000, 2000 to 4000, 4000 to 6000, and >6000 s. (**E** and **F**) Distributions of interfacial tension and bending rigidity values as measured within the four time domains indicated in (D). The colors of the histograms match the bars in (D), except where all four histograms overlap, which has been left white for clarity. The histograms clearly show a large overlap.

Another pertinent question is the role of time in the population of stress granules and how their properties are affected. Condensates are known to mature over time, and previous research suggests that the terminal state is likely to be a single-mode Maxwell fluid (*18*). Figure 6 (A to C) corresponds to typical images of observed stress granules at various time stamps. Here, *t* = 0 is when the cells are induced with sodium arsenite. Visually, it is clear that, with increasing time, there are larger and more elongated granules. However, as we have argued in Fig. 4, these are the typical granules that have matured and therefore filtered out during the flicker spectroscopy analysis. To provide more quantitative insights, in Fig. 6 (D to F), we have divided our sodium arsenite–induced stress granules data into four time domains: 0 to 2000, 2000 to 4000, 4000 to 6000, and >6000 s. The fraction of stress granules that pass the filter decreases with time, signifying the presence of large elongated mature granules in addition to large spheroid granules that pass the filter and can be analyzed. The measured interfacial tension and bending rigidity distributions, not just the average values, are consistent across time and can be well characterized, as shown in Fig. 6 (D to F). We show in fig. S5 that there is no correlation of time with interfacial tension, bending rigidity, or granule size. Together, this indicates that those granules that pass the filter have consistent characteristics across time.

### Comparing different types of stress granules

**Fig. 7.**
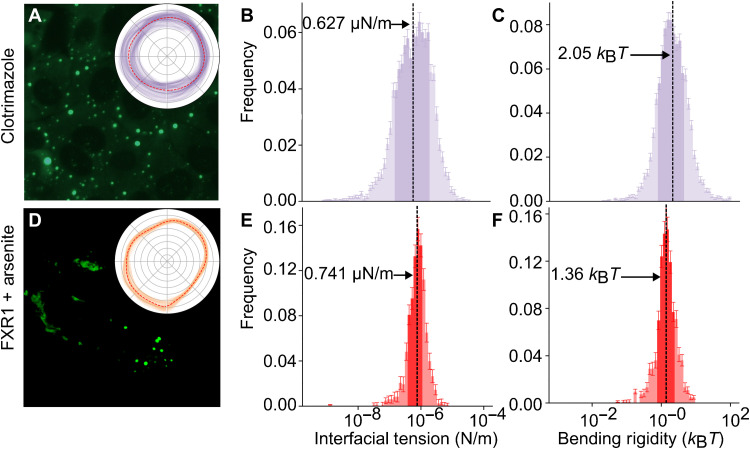
Experimental results for cells treated with clotrimazole (top row) and for cells transfected with FXR1-YFP and treated with sodium arsenite (bottom row). Overview images of the cells 30 min after treatment are shown in the left column (**A** and **D**), the distribution of interfacial tensions across the population of analyzable granules is shown in the middle column (**B** and **E**), and similar distributions for the bending rigidity are on the right (**C** and **F**). Insets in the left column show the variation of the boundary of a representative granule over the course of the experiment, sampled every 10 ms. The red dashed line shows the average position of the boundary. On each histogram, the geometric mean is marked by a vertical line, annotated with its value. In addition, each histogram has a darker region, in which 67% of the granules are found. The choice of treatment affects the mechanical properties of the granules. Clotrimazole granules have a notably lower interfacial tension than sodium arsenite granules and a much wider spread of interfacial tensions. Farnesoid X receptor 1 (FXR1) granules have an even narrower spread and an interfacial tension similar to sodium arsenite. Last, FXR1 granules have a substantially lower bending rigidity on average, indicating a change in the structure at the interface.

Having shown that the material properties of sodium arsenite–induced stress granules are consistent across time and can be well characterized, we seek to compare the interfacial tension and bending rigidity of stress granules induced using 20 μM clotrimazole instead of 200 μM sodium arsenite. Visually, clotrimazole-induced granules (Fig. 7A) are morphologically distinct, appearing, on average, smaller and more rounded than sodium arsenite–induced granules (Fig. 4A). The insets in Figs. 4A and 7A provide examples of the collected granule boundaries across the entire measurement period, sampled every 10 ms. For the clotrimazole-induced granules, we initially record 41,573 granules across several independent experiments. Following the same filtering steps that we used for the sodium arsenite case, 14,244 granules pass the outline filter, 13,965 granules pass the bending rigidity filter, and 4556 granules pass the error fitting filter.

As already highlighted in Fig. 4, in each case, the interfacial tension and bending rigidity histograms have a log normal–type distribution with broad tails. The observed values typically span across two to three orders of magnitude, but as summarized in Table 1, 67% of the granules (equivalent to 1 SD in a log normal distribution) is confined within an order of magnitude. Comparing the average values, clotrimazole granules (0.627 μN/m) have a lower interfacial tension than sodium arsenite granules (1.26 μN/m), but with comparable bending rigidity (2.05 *k*_B_*T* for clotrimazole versus 2.08 *k*_B_*T* for sodium arsenite). This difference in interfacial tension is visible in the breadth of the interfacial fluctuations (compare the insets in Figs. 4A and 7A). The correlation relationships for clotrimazole granules are qualitatively similar to those seen in Fig. 5 for sodium arsenite granules (see fig. S6).

**Table 1.** Summary of the mechanical properties of stress granules under various treatments. Geometric averages of the interfacial tension and bending rigidity for sodium arsenite and clotrimazole granules as well as for those with farnesoid X receptor 1 (FXR1)–overexpressing U2OS cells. The ranges of values that contain 67% of the granules, equivalent to 1 SD in a log normal distribution, are also provided.

Interfacial tension	Geometric mean	±1 SD in log normal distribution
Sodium arsenite	1.26 μN/m	0.530–3.08 μN/m
Clotrimazole	0.627 μN/m	0.171–2.33 μN/m
FXR1 overexpression and sodium arsenite	0.741 μN/m	0.430–1.33 μN/m
**Bending rigidity**	**Geometric mean**	**±1 SD in log normal distribution**
Sodium arsenite	2.08 *k*_B_*T*	0.934–4.80 *k*_B_*T*
Clotrimazole	2.05 *k*_B_*T*	0.816–5.2 *k*_B_*T*
FXR1 overexpression and sodium arsenite	1.36 *k*_B_*T*	0.828–2.54 *k*_B_*T*

Similarly, we compare changes in mechanical properties as a result of altered stress granule composition by comparing sodium arsenite–induced granules in control U2OS cells and farnesoid X receptor 1 (FXR1)-overexpressing U2OS cells (*20*). FXR1 is a constituent of stress granules, colocalizes with G3BP1, and has been reported to be overexpressed in cancer (*46*). Several groups have suggested that there are heterogeneities in the composition at the bulk and the interface of stress granules, and this can be modulated by the stoichiometry of the components such as G3BP and FXR1 (*20*, *21*). We hypothesize that compositional modulation and resulting rearrangement of stress granule components would be expected to alter condensate mechanical properties potentially detectable by flicker spectroscopy. We capture 28,899 granules over several independent experimental runs and select 830 granules for analysis using the same filtering as described above.

FXR1 overexpression results in constitutively formed large irregularly shaped G3BP1- and FXR1-positive foci, which when treated with sodium arsenite form spheroid stress granules, likely due to the increased availability of cytoplasmic RNA following sodium arsenite–induced translational arrest, as shown in Fig. 7D. Compared to sodium arsenite–induced granules in control cells, the FXR1-overexpressing cells have a lower interfacial tension (0.741 μN/m) and bending rigidity (1.36 *k*_B_*T*) (see Fig. 7, E and F). Once again, we see no qualitative difference in the correlations between properties of simple sodium arsenite granules and those treated with FXR1 (see fig. S7).

## DISCUSSION

In conclusion, we have developed a high-throughput flicker spectroscopy pipeline optimized for exploring the mechanical properties of condensate organelles in cellulo under physiological conditions. Flicker spectroscopy allows us to investigate two important condensate properties: interfacial tension and bending rigidity. Interfacial tension regulates fusion and growth of droplets as well as determines how condensates form hierarchical structures (droplet in droplet, droplet next to droplet, etc.) (*2*, *47*, *48*) and how condensates interact with other cellular components, such as lipid membranes (*11*, *16*, *49*), DNAs (*50*–*52*), and cytoskeletal filaments (*12*, *53*). Bending rigidity is a measure of stiffness of the interface, which we show to be an essential feature of stress granules.

Conceptually, the high-throughput in cellulo flicker spectroscopy approach presented here further enables a number of key insights. The fact that we need to correct for the condensate base shape and include bending rigidity indicates that stress granules are not simple liquids and supports the view that stress granules are viscoelastic (*40*, *54*, *55*). Our analysis also shows that each type of stress granule has a broad distribution of mechanical properties. This means that any method or study that considers a small number of granules (or, more generally, condensates) may not detect some subtle differences between granules. In contrast, our high-throughput framework allows us to compare distributions of large populations of stress granules under physiological conditions. Pleasingly, such large-scale surveys show that condensate material properties can be well characterized and measurable across time. We find neither interfacial tension nor bending rigidity correlates with size, but interfacial tension has a dependence on condensate shape while bending rigidity does not. They also allow us to distinguish stress granules induced by different approaches or under different stoichiometries of constituent proteins.

In this study, we have demonstrated a drop in interfacial tension in stress granules formed due to clotrimazole treatment compared to sodium arsenite–induced granules. These granules are morphologically distinct and have clearly distinguishable fluctuation spectra and interfacial tensions. Specifically, clotrimazole granules have a more spherical form but a lower surface tension. Again, such observation better aligns with the view that stress granules are viscoelastic, where the material properties are determined by a combination of viscous and elastic moduli. It is likely that the different chemical stresses result in the formation of granules with different compositions. Moreover, we have seen that bending rigidity is altered in FXR1-overexpressing cells. This corroborates published studies showing that altered stoichiometry of G3BP1 and FXR1 results in rearrangements of molecules at the interface of stress granules (*20*, *21*). It has been shown that molecules with surfactant-like properties can adsorb to the interface of condensates (*13*, *19*, *56*). The presence of such molecules will lead to an elastic component on the condensate interface, and the compression of such an interface may explain the origin of the bending rigidity that we observed. Furthermore, we speculate that the resulting stresses at the interface can be anisotropic, and this may, in turn, favor a nonspherical granule shape.

We believe that a number of key advantages of live-cell flicker spectroscopy could be harnessed in future work, for example, to evaluate the changes in mechanical properties of different condensates in living cells as a function of size, time, and location inside the cell, in the presence of disease relevant mutations, or in response to candidate drugs (*57*). It will also be interesting to simultaneously measure interfacial tension of different types of condensates in proximity by using dual-labeled cells expressing fluorescently labeled marker proteins for each compartment, such stress granules and P-bodies, which are known to coexist, exchange material but retain their identity.

This method, however, is not free from challenges and limitations, and there remains a number of exciting avenues to refine and extend our analysis. In terms of technical measurement challenges, the high number of microscope frames required means that users must be careful that bleaching is not distorting the outline of granules over time. It is also important to ensure that the granules are imaged in the equatorial plane (see section SM3 in the Supplementary Materials). In the future, we can further consider correcting for optical effects that can contribute to imprecision in measuring the rigidity for small objects (*58*). In terms of data analysis, we currently need to filter out very small granules and mature granules that have become too elastic or gel like; hence, we primarily have access to granules during the intermediate, liquid-like stage of their life cycle. It will be interesting to infer condensate viscosity/viscoelasticity from the temporal variation of fluctuation modes, which would allow us to extend our analysis to more mature droplets. Another avenue of interest is to consider possible effects of active mechanical forces. For example, recent studies have shown that chemical reactions within condensates can affect properties such as their growth rate (*59*). Thus far, our analysis is based on equilibrium statistical physics, supported by the good fit between the measured and predicted spectra. Previous works on active membranes (*60*, *61*) have shown that the presence of active forces is accompanied by clear deviation of the spectra from the equilibrium predictions, which we do not see here. However, this does not completely exclude active mechanical forces whose effects primarily rescale the effective temperature of the system (*62*).

## MATERIALS AND METHODS

### Cell culture

The U2OS cell line ΔΔ17-U2OS-EGFP-G3BP was a gift from N. Kedersha (*38*). These cells have endogenous G3BP1 and G3BP2 removed and the fluorescently labeled GFP-G3BP1 added. Cells were routinely passaged in T75 and T25 flasks containing Dulbecco’s modified Eagle’s medium (DMEM) (Sigma-Aldrich, no. D5671) with 10% fetal bovine serum (FBS; Sigma-Aldrich, no. F7524-500ML). The cells were stressed in 35-mm glass dishes (Ibidi, cat. no. 81158) for imaging. Here, we use two frequently used chemicals to induce stress granules, sodium arsenite and clotrimazole (*63*, *64*). Sodium arsenite granules were induced by treating cells in 35-mm dishes with sodium arsenite (Sigma-Aldrich, no. 1062771000) in DMEM at a concentration of 200 μM for the time indicated. Stress granules typically appeared from 15 to 30 min onwards. The data for cells treated with sodium arsenite were collected in two batches on 2 days. Data for other treatments were collected in one batch. Clotrimazole treatment was carried out at a concentration of 20 μM clotrimazole (Sigma-Aldrich) in 0% FBS Opti-MEM (Thermo Fisher Scientific, no. 31985070) (all treatments). Opti-MEM alone does not induce granule formation. The FXR1-YFP plasmid was a gift from N. Kedersha (*20*). U2OS cells were transfected with 500 ng of FXR1 using Lipofectamine (Thermo Fisher Scientific, no. 18324012). Fixed cells were prepared by washing twice with 37°C PBS (Sigma-Aldrich, no. D8537-500ML), fixing in 4% paraformaldehyde (Thermo Fisher Scientific no. 28908) for 10 min, and again washing twice with 37°C PBS.

### Microscopy

Andor Dragonfly 505 spinning disk confocal microscope was used with a 100× 1.49 numerical aperture (NA) CFI SR HPApo total internal reflection fluorescence oil immersion lens with a ×1.5 magnification objective (Nikon MRD01995) and a 60× 1.40 NA CFI apochromat Lambda oil immersion lens with a ×2 magnification objective (Nikon MRD71670) with a 1024-by-1024 pixel iXon 888 Life electron-multiplying charge-coupled device camera, with a read noise of <1. Using finite burst mode, we collect 1000 frames on each field of view for 40 s with an exposure time of 10 ms. Illumination mode Power Density 2 (PD2) was used. We used a laser power of 10 to 20% and a gain of around 152 to keep bleaching below 30%. The signal-to-noise ratio was ~5 in both the condensates and the background.

### Image analysis

In the microscopy images, stress granules appear as bright bodies against a darker background, as can be seen in Fig. 1C. Some G3BP-GFP remains in the cytoplasm as granules form; hence, the background is not completely dark and the strength of the signal varies from cell to cell, which makes simple thresholding an unsuitable method of granule detection. Therefore, we use the “difference-of-Gaussian” approach, which is better able to detect locally correlated features against a complex background by searching for local maxima, as shown in Fig. 1D (*25*). This gives the coordinates of the center point of each granule in the image. We remove any granule with an area less than 100 pixels, as these are too small for our analysis. We estimate the area using a flood fill, as shown in Fig. 1E, with a threshold of 0.5 *l*_max_,where *l*_max_ is the maximum intensity in the image.

A bespoke edge detection algorithm is used to detect the boundary of each granule on the imaging plane, as illustrated for an example granule in Fig. 1F. Granules are imaged through the equatorial plane, and since the cells are quite flat, *z* stack confocal images of fixed cells show that the granules broadly occupy the same plane (see section SM3 in the Supplementary Materials), justifying the assumption that the granules do not diffuse into and out of the plane over the course of our measurements to such an extent that they affect our results. In section SM3 in the Supplementary Materials, we also simulate fluctuating stress granules, which are then analyzed at different *z* planes. We found that the results are robust, with potential deviations of the imaging plane from the granule equator. Because any typical deviation is within 20°, we show that the corresponding errors are small compared to the typical distribution in the values of interfacial tension and bending rigidity.

We express the boundary of a granule as the distance *D*(φ) from the center point to the edge as a function of angle φ for a set of 400 angles evenly distributed between 0 and 2π. The edge is the point of maximum directional gradient of the fluorescent signal $g=\nabla I\cdot \hat{r}$, where *I* is the image intensity and $\hat{r}$ is the radial unit vector. The gradient at the pixel in row *i*, and column *j*, ∇*I*_*i*,*j*_, is calculated using a fourth-order approximation in the *x* and *y* directions

$$\nabla I_{i,j}=\frac{1}{12}(I_{i-2,j}-8I_{i-1,j}+8\;I_{i+1,j}-I_{i+2,j},I_{i,j-2}-8I_{i,j-1}+8\;I_{i,j+1}-I_{i,j+2})$$ (5)

Harnessing the information on *D*(φ), we reject any boundary where there is a large discontinuous jump between two adjacent points. This is often due to granule merging events or when a granule wets around a subcellular surface, leading to overhangs where the granule cannot be represented by a radial function. Examples of such cases are shown in fig. S3. These rejections are evaluated on a frame-by-frame basis, giving a boundary pass rate for each granule, and we remove a granule from further analysis if the boundary is rejected in more than 60% of the frames. Stress granule boundaries are collected every 10 ms across the entire measurement period to yield a fluctuation spectrum per granule for thousands of stress granules.

The subpixel boundary detection technique that we use can detect fluctuations as small as 0.1 pixel (see section SM4 in the Supplementary Materials) (*37*). We observe that the average amplitude of the 15th fluctuation mode is 0.14 pixels, close to the limit of what we can reliably resolve. Therefore, we only fit the first 15 fluctuating modes.

### Simulations of rotating rigid bodies

We generate a simulated, quenched rigid body with a normalized interface deformation given by

$$u(\theta ,\varphi ,t)=\sum_{l=2}^{15}\sum_{m=-l}^{l}Y_{lm}(\theta ,\varphi )U_{lm}e^{i\theta^{\prime }}e^{i\varphi^{\prime }}$$ (6)

where *U_lm_* is a random magnitude between 0 and 1 selected from the pink noise spectrum and (θ, φ) is a random point on the unit sphere, where θ is the angle of inclination and φ is the azimuth angle. The radius of the simulated droplet is normalised to 1 in the simulation unit. The choice of pink noise, instead of uniform (white) noise, ensures that the magnitude of the fluctuation decreases with *l*, yielding more physically plausible shapes when compared to stress granules. The rigid body then undergoes a series of 100 random rotations. First, a random axis is generated, uniformly distributed on the unit hemisphere. Then, a random rotation magnitude is selected from a normal distribution with a mean of 0 and an SD of 2^β^. The equatorial cross section is taken after each rotation in the series and is subjected to the fluctuation spectrum analysis akin to the experimental data. As shown in Fig. 3B, this yields an estimate of the spectrum that would be measured for a rough rigid body undergoing random rotations.

## Supplementary Materials

### This PDF file includes:

Supplementary Text SM1 to SM4 Figs. S1 to S8 References

## Notes

View/request a protocol for this paper from *Bio-protocol*.
