# Supplementary material for: A bending rigidity parameter for stress granule condensates
Source: Sci Adv. 2023 May 17;9(20):eadg0432. doi: 10.1126/sciadv.adg0432 (PMC10191439; doi:10.1126/sciadv.adg0432)
Supplement: Supplementary file 1 — Supplementary Text SM1 to SM4 Figs. S1 to S8 References [file sciadv.adg0432_sm.pdf]

Science Advances
AAAS

Supplementary Materials for

A bending rigidity parameter for stress granule condensates

Jack O. Law *et al.*

Corresponding author: Halim Kusumaatmaja, halim.kusumaatmaja@durham.ac.uk;
Sushma N. Grellscheid, sushma.grellscheid@uib.no

Sci. Adv. **9**, eadg0432 (2023)
DOI: 10.1126/sciadv.adg0432

This PDF file includes:

Supplementary Text
SM1 to SM4
Figs. S1 to S8
References

Supplementary Text

SM1. Derivation of the theoretical spectrum.

In this section we discuss the derivation of the theoretical spectrum given in equation (3) of the main text. The interface of a granule, S, is described as a perturbation, u, on the interface of a sphere radius R,

$$S(\theta, \varphi, t) = R[1 + u(\theta, \varphi, t)]. \tag{S1}$$

We may then write the perturbation as a weighted sum of spherical harmonics Y_{lm},

$$u(\theta, \varphi, t) = \sum_{l=2}^{\infty} \sum_{m=-l}^{l} Y_{lm}(\theta, \varphi) U_{lm}(t), \tag{S2}$$

where $U_{lm}(t)$ is the magnitude of the given spherical harmonic term. l and m are the indices of the spherical harmonic modes. In general, $l \geq 0$ and $-l \leq m \leq l$. The $l = 0$ mode corresponds to a uniform (spherical) growth and shrinking. The time-average of this mode simply gives the average radius of the stress granule, which should be constant for a fixed-volume granule. It is represented by the first term in equation (S1). The $l = 1$ mode represents translations of the whole granule, so has no bearing on thermal fluctuations of the interface. Therefore, we can discard the terms corresponding to $l = 0$ and 1 modes.

Assuming the energies to deform the condensate are as given by the Helfrich Hamiltonian, the average energy for each fluctuation mode is given by (*65, 66*)

$$\langle E_{lm} \rangle = \frac{\kappa}{2} \left| U_{lm} \right|^2 (l + 2)(l - 1)[l(l + 1) + \bar{\sigma}], \tag{S3}$$

where $\bar{\sigma} = \sigma R^2/\kappa$ is the dimensionless interfacial tension, with R the mean condensate radius. The equipartition theorem states that all these modes should have the same time-averaged energy $\langle E_{lm} \rangle = k_B T/2$, giving an expected spectrum of

$$\langle \left| U_{lm} \right|^2 \rangle = \frac{k_B T}{\kappa} \frac{1}{(l+2)(l-1)[l(l+1) + \bar{\sigma}]}. \tag{S4}$$

In practice, we are only able to measure a two-dimensional slice of the interface, so we must relate the three-dimensional fluctuation modes $\langle \left| U_{lm} \right|^2 \rangle$ into terms that we can observe directly. As we assume that the granules are imaged through the equatorial plane of the granule, then we can see a cross-section of the shape perturbations $\hat{u}(\varphi, t) = u(\pi/2, \varphi, t)$ without loss of generality, corresponding to the $\theta = \pi/2$ plane.

We can relate $\hat{u}$ to the two-dimensional fluctuation spectrum, by writing it as a sum of Fourier modes

$$\hat{u}(\varphi, t) = \sum_{q=2}^{\infty} \nu_q e^{-iq\varphi},$$

(S5)

where ν_q is the amplitude for mode q. Using standard Fourier transform, we can calculate ν_q via

$$\nu_q(t) = \frac{1}{2\pi}\int_0^{2\pi} \hat{u}(\varphi, t) e^{iq\varphi} d\varphi.$$

(S6)

Using the definition in equation (S2) for the condensate fluctuation and taking its projection for the $\theta = \pi/2$ plane, we obtain

$$\nu_q = \frac{1}{2\pi}\int_0^{2\pi} \sum_{lm} U_{lm} Y_{lm}(\pi/2, \varphi) e^{iq\varphi} d\varphi,$$

$$\nu_q = \frac{1}{2\pi}\int_0^{2\pi} \sum_{lm} U_{lm} N_{lm} P_{lm} \cos(\pi/2) e^{im\varphi} e^{iq\varphi} d\varphi,$$

(S7)

where P_{lm} and N_{lm} are the associated Legendre polynomials and normalisation factor

$$N_{lm} = \sqrt{\frac{2l+1}{4\pi}\frac{(l-m)!}{(l+m)!}},$$

(S8)

respectively. Solving equation (S7) (*66, 67*) gives a relation between the time averaged magnitudes of the observable fluctuations ν_q and the full three-dimensional fluctuations U_{lm}

$$\langle |\nu_q|^2 \rangle = \sum_{l=q}^{l_{max}} \langle |U_{lq}|^2 \rangle N_{lq}^2 P_{lq}^2(\cos \pi/2).$$

(S9)

We take $l_{max} = 75$ to ensure a good convergence (*66, 68*). The result is usually accurate to within 0.1% after 40 terms in l. Substituting equation (S4) into equation (S9), we obtain

$$\langle |\nu_q|^2 \rangle = \frac{k_B T}{\kappa} \sum_{l=q}^{l_{max}} \frac{N_{lq}^2 P_{lq}^2(0)}{(l+2)(l-1)[l(l+1)+\bar{\sigma}]},$$

(S10)

which is the predicted spectrum of the experimentally observable fluctuations.

SM2. Spectrum fitting.

In flicker spectroscopy, the final step in the analysis to extract the values of σ and κ is to find the best fit between the theoretical and measured spectra.

We have to be careful in the choice of how we define the best fit. An error function, $\varepsilon(\sigma, \kappa)$, gives a quantitative value for the quality of the fit. A poor choice of error function leads to an over-emphasis of certain parts of the spectrum. For example, one possibility is to choose a root mean squared approach with

$$\varepsilon^2(\sigma, \kappa) = \sum_q \left(\left|F_{q,theo}\right|^2 - \left|F_{q,exp}\right|^2 \right)^2,$$

(S14)

where $\left|F_{q,theo}\right|^2$ is the theoretical spectrum given by equation (S10) and $\left|F_{q,exp}\right|^2$ the measured spectrum either with or without the correction given in Eqs. 4 and 5 of the main text. This choice of error function leads the minimiser to prioritise only the first orders (low q) and neglect higher order terms that typically have much smaller fluctuation amplitudes. This leads to a very poor overall fit, as shown in Fig. S1. We improve this by introducing an error measure that is based on the error ratio between the values of theoretical spectrum $\left|F_{q,theo}\right|^2$ and the measured spectrum $\left|F_{q,exp}\right|^2$,

$$\varepsilon_{log} = \sum_q \left| log_{10}\left[\frac{\left|F_{q,theo}\right|^2}{\left|F_{q,exp}\right|^2} \right] \right|.$$

(S15)

We use a combination of parameter sweeps and L-BFGS-B algorithms (*69*) to minimise the fitting error, ε_{log}.

After employing the error function in equation (S15), we find some granules nevertheless have a poor fit. Many of these granules are simply too small or too short-lived to yield a reliable spectrum. For others, the error may be due to the granule having undergone ageing and become more solid-like, or lying far from the equatorial plane (see SM3). We filter out these granules by removing any granule with a best fit worse than $\varepsilon_{log} = 0.5$. This cut-off is chosen as beyond this point the theoretical and experiential spectra show noticeable deviations from one-another.

SM3. Potential Uncertainties in Identifying the Equatorial Plane.

The calculation in **SM1** makes the assumption that the stress granules are imaged through the equatorial plane. Here we will test this assumption. During microscopy, we select an imaging plane which maximises the area of the visible granules, which would correspond to the equatorial plane if all the granules are co-planar. To test this, we took a z-stack confocal image of a number of fixed cells and inspected slices through them. The typical result, as seen in Fig. S2A, shows that the granules are approximately co-planar, due to the fact that the thickness of the cells are similar to the granule diameter. We quantified this by identifying the best image-plane and calculating how this plane compares to the equatorial plane of each individual stress granule (see Fig. S2B). Typically, we find the image plane and equatorial plane are within 20°, where the divergence is calculated as $\alpha = sin^{-1}(d/R)$, where d is the distance between the image-plane and granule's equatorial plane and R is the granule radius.

We next analyse the effect this divergence will have on the estimate of interfacial tension and bending rigidity by simulating granules, taking slices at different planes, and subjecting these slices to our fitting analysis.

Granules are simulated as follows. We generate fluctuating granules with an interface defined by

$$u(\theta, \varphi, t) = \sum_{l=2}^{\infty} \sum_{m=-l}^{l} Y_{lm}(\theta, \varphi) \langle \left|U_{lm}\right|^2 \rangle e^{i\varphi'} e^{i\theta'} e^{i\omega t},$$

(S16)

where φ' and θ' are random phases chosen at each time-step and ω is a frequency chosen to be equal to the frame-rate. $\langle |U_{lm}|^2 \rangle$ is obtained from the theoretical spectrum given in equation (S4). For concreteness, we use $\kappa = 0.5 k_B T$, $R = 1 \mu m$, and the interfacial tension is varied over the range $\sigma = 0.1 - 10 \mu N/m$.

Fig. S2C shows the change in the measured interfacial tension of the granule as the image plane moves away from the equator. A deviation of 20° typically causes a <20% change in the measured value. For the bending rigidity, shown in Fig. S2D, the change is on average <3%, although this varies more than the interfacial tension depending on the granule parameters (it can be as high as 6%). Both of these are small compared to the spread of values reported in Table 1 of the main text.

SM4. Sub-Pixel Image Analysis

The edge detection method described in *materials and methods* is able to take advantage of the image gradient to detect fluctuations of up to 0.1 pixel. To demonstrate this, we simulate an interface in one dimension using

$$I(x) = \frac{1}{2}\left[tanh\left(\frac{x - x_0}{\varsigma}\right)\right], \qquad (S17)$$

where $I(x)$ is the image intensity along the x direction, x_0 is the position of the interface, and ς is the interface width. We find $\varsigma = 0.8$ closely approximates the interface of a granule. Figure S8A shows two such interfaces, with $x_0 = 20.0$ and $x_0 = 20.1$, sampled at integer values of x to represent individual pixels. Figure S8B shows the gradient calculated from these samples, using equation 6 (in one dimension), with a cubic interpolation. The peaks in the gradients can be separated from each other, demonstrating a resolution of 0.1 pixel.

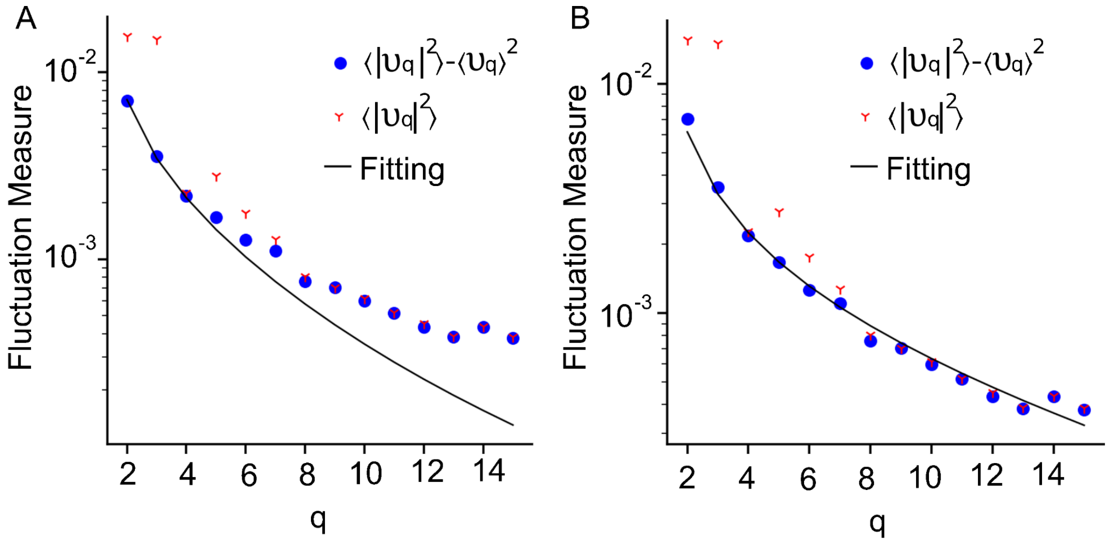

Fig S1. Comparison of fitting methods. (A) An example spectrum fit using equation (S14).The fit is only good for the first few modes. (B) The same spectrum fit with equation (S15). The fit is good for all modes.

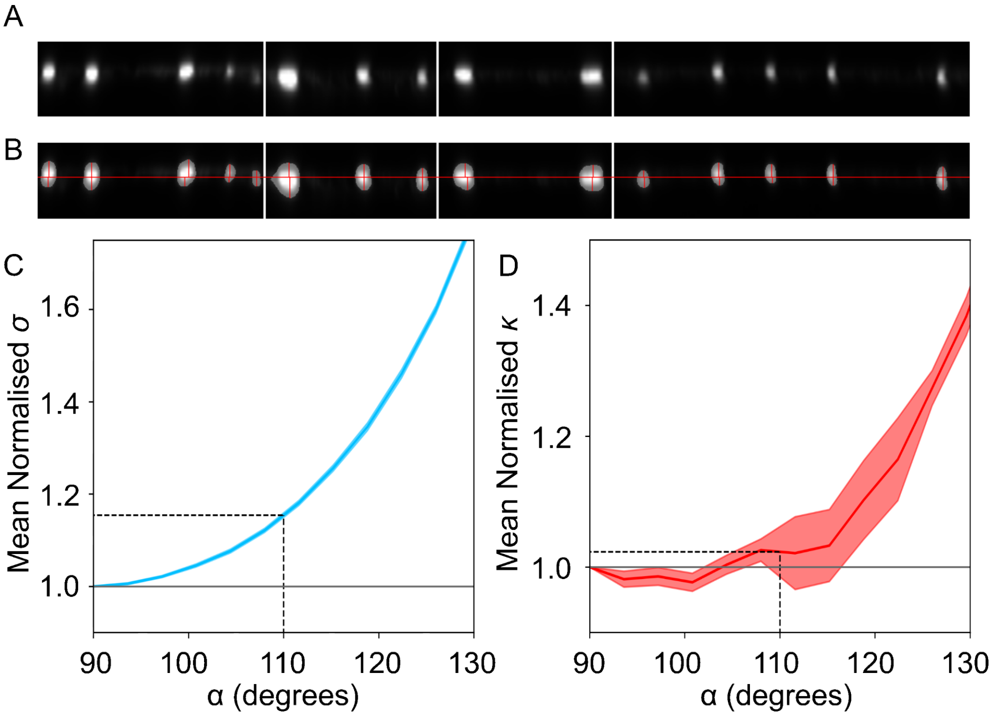

Fig S2. The effect of the deviation of the image plane from the granule equator. (A) Slices through 4 different cells taken from a confocal Z-stack image of fixed cells, on a single plate, treated with sodium arsenite. It can be seen that the stress granules are roughly co-planar both within a cell and across the population of cells. (B) A threshold is applied to the image. The best imaging plane is shown as a red horizontal line and the vertical red lines show the top and the bottom of each granule that intersects the imaging plane. The deviation between the imaging plane and the granule equator is typically within 20°. (C) and (D) We pass simulated granules, sliced at different planes, to the spectrum fitting algorithm. A deviation from the equator of 20° leads to an error of about 20% in interfacial tension and 3% in bending rigidity (dashed lines). This is well below the range of values reported in Table 1.

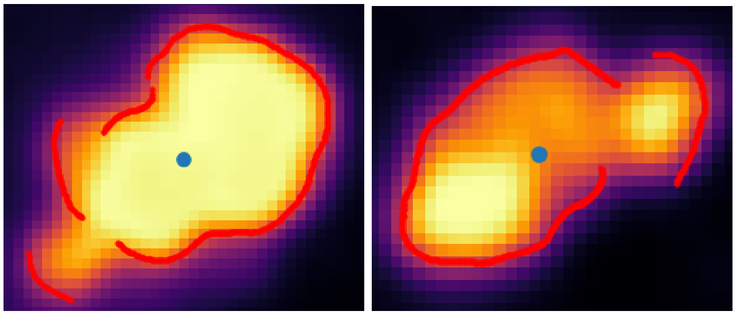

Fig S3. Examples of imaged granules that fail the shape filter. Typically, we find large discontinuous jumps between adjacent points, and the granules cannot be represented by a radial function. These could be due to granule merging events or when a granule wets around a sub-cellular interface.

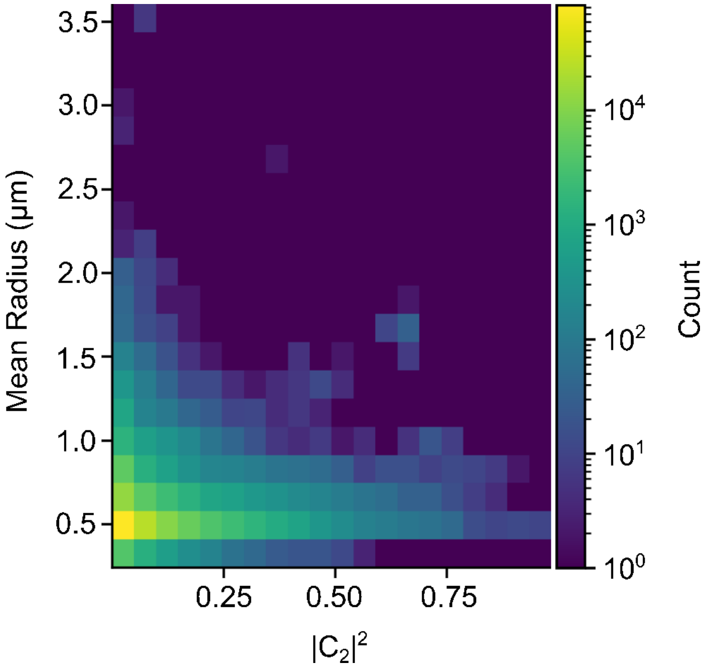

Fig S4. **Distribution of granules as a function of their mean radius and, prior to any filtering.** Compared to Fig. 5A, it is clear that our filtering protocols remove large, highly non-circular granules. These are likely to represent mature, solid-like granules, which are unsuitable for Flicker Spectroscopy.

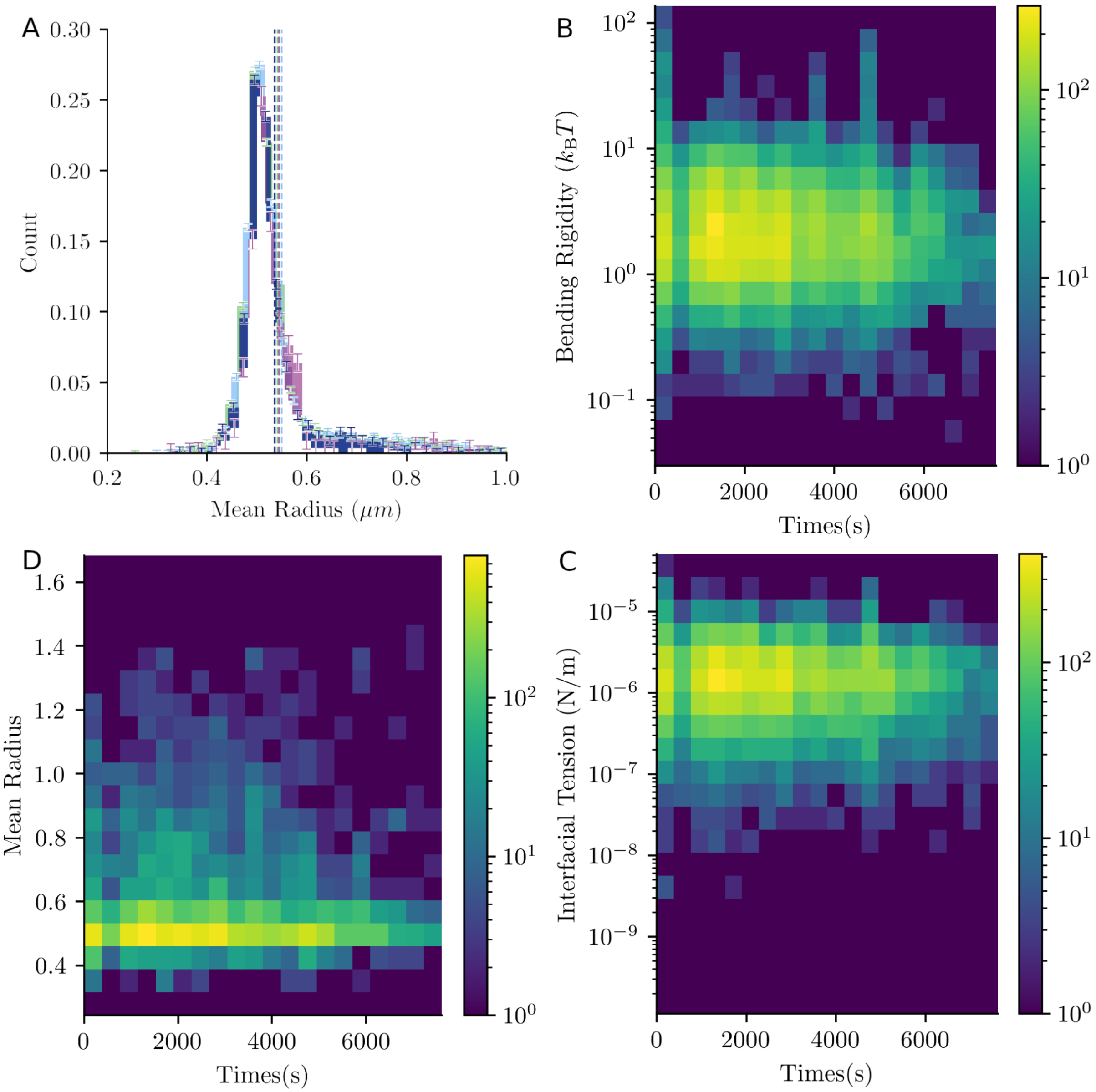

Fig S5. The relationship between the properties of granules induced by sodium arsenite and time. (A) Histograms of the mean radius for the four time-bands used in figure 6. The colour scheme is retained. (B-D) The distribution of the granules as a function of time and interfacial tension, bending rigidity and mean radius. There seems to be no systematic variation of the physical quantities on these timescales.

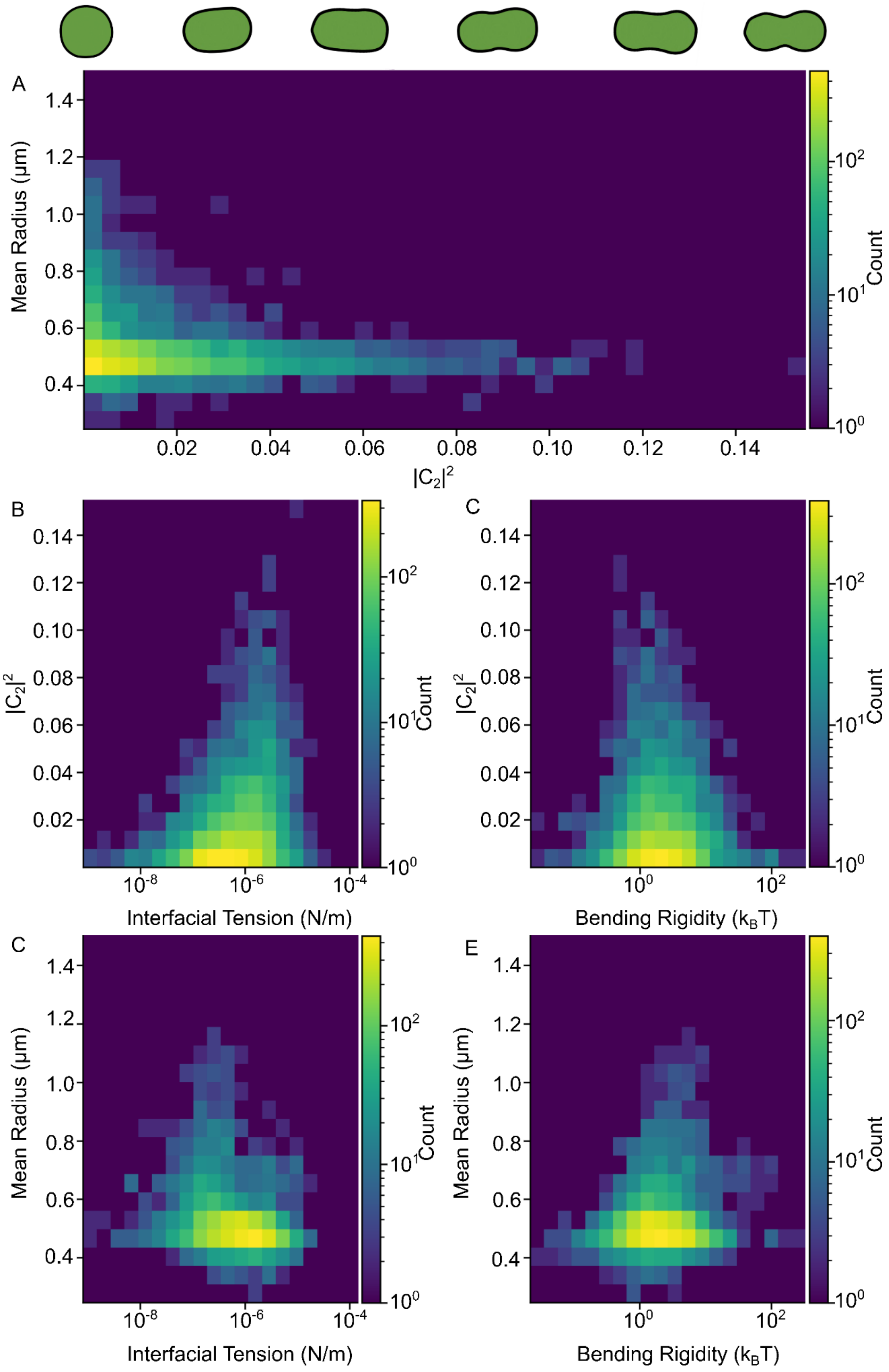

A
Mean Radius (μm)
$|C_2|^2$
Count
B
C
Interfacial Tension (N/m)
Bending Rigidity (k_BT)
E

Fig S6. The relationships between clotrimazole granule properties. The distribution of Clotrimazole granules as a function of their mean radius, circularity measure ($|C_2|^2$), interfacial tension and bending rigidity. We observe similar qualitative behaviour to Fig. 5 of the main text for sodium arsenite induced stress granules.

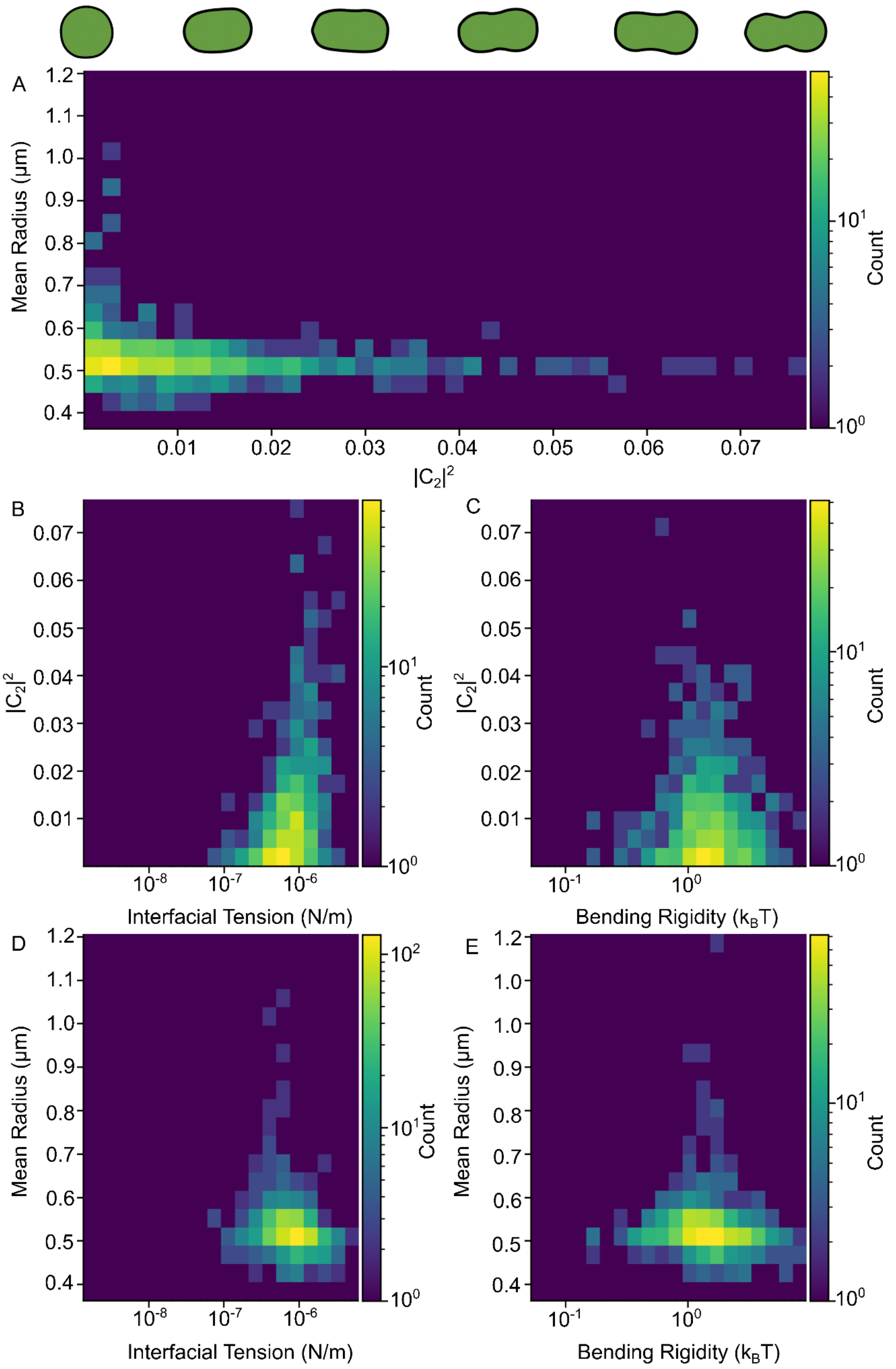
A
Mean Radius (μm)
|C2|^2
Count
B
Interfacial Tension (N/m)
C
Bending Rigidity (kBT)
D
E

Fig S7. The relationships between sodium arsenite granule properties in cells overexpressing FXR1. The distribution of granules in FXR1 overexpressing U2OS cells as a function of their mean radius, circularity measure, interfacial tension and bending rigidity. We observe similar qualitative behaviour to Fig. 5 of the main text for control U2OS cells.

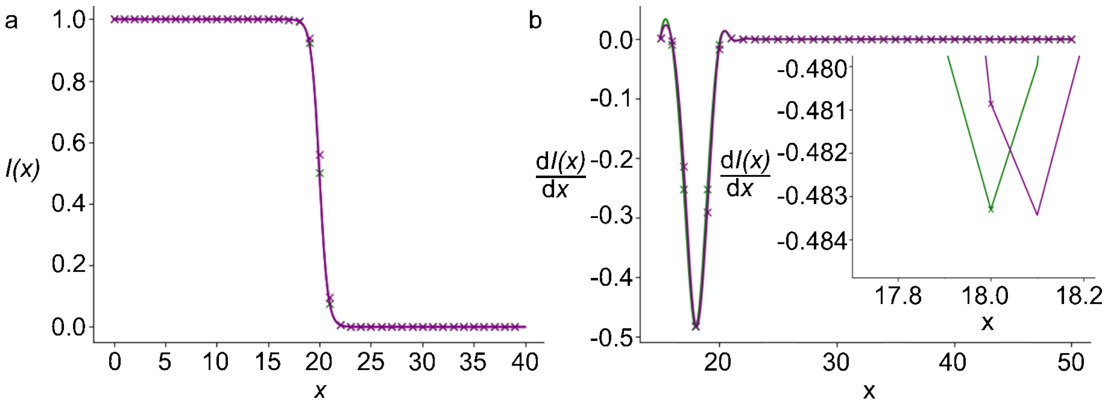

Fig S8. Verification of the sub-pixel resolution of the boundary detection method. (A) Two simulated interfaces with $x_0 = 20.0$ (green) and $x_0 = 20.1$ (purple). Crosses show the interfaces sampled at one-pixel intervals. (B) The gradient of the interface at each pixel, as calculated with equation (6) (crosses). The solid line shows a cubic interpolation between these points. The inset shows a magnification around the minimum. Using this interpolation, we can distinguish the minima of each interface.

REFERENCES AND NOTES

1. C. P. Brangwynne, T. J. Mitchison, A. A. Hyman, Active liquid-like behavior of nucleoli determines their size and shape in *Xenopus laevis* oocytes. *Proc. Natl. Acad. Sci. U.S.A.* **108**, 4334–4339 (2011).

2. M. Feric, N. Vaidya, T. S. Harmon, D. M. Mitrea, L. Zhu, T. M. Richardson, R. W. Kriwacki, R. V. Pappu, C. P. Brangwynne, Coexisting liquid phases underlie nucleolar subcompartments. *Cell* **165**, 1686–1697 (2016).

3. L. Galganski, M. O. Urbanek, W. J. Krzyzosiak, Nuclear speckles: Molecular organization, biological function and role in disease. *Nucleic Acids Res.* **45**, 10350–10368 (2017).

4. C. L. Riggs, N. Kedersha, P. Ivanov, P. Anderson, Mammalian stress granules and P bodies at a glance. *J. Cell Sci.* **133**, jcs242487 (2020).

5. H. Wu, M. Fuxreiter, The structure and dynamics of higher-order assemblies: Amyloids, signalosomes, and granules. *Cell* **165**, 1055–1066 (2016).

6. C. Mathieu, R. V. Pappu, J. P. Taylor, Beyond aggregation: Pathological phase transitions in neurodegenerative disease. *Science* **370**, 56–60 (2020).

7. S. F. Banani, H. O. Lee, A. A. Hyman, M. K. Rosen, Biomolecular condensates: organizers of cellular biochemistry. *Nat. Rev. Mol. Cell Biol.* **18**, 285–298 (2017).

8. T. Mittag, R. V. Pappu, A conceptual framework for understanding phase separation and addressing open questions and challenges. *Mol. Cell* **82**, 2201–2214 (2022).

9. S. F. Shimobayashi, P. Ronceray, D. W. Sanders, M. P. Haataja, C. P. Brangwynne, Nucleation landscape of biomolecular condensates. *Nature*, **599**, 503–506 (2021).

10. L.-P. Bergeron-Sandoval, S. W. Michnick, Mechanics, structure and function of biopolymer condensates. *J. Mol. Biol.* **430**, 4754–4761 (2018).

11. S. Botterbusch, T. Baumgart, Interactions between phase-separated liquids and membrane surfaces. *Appl. Sci. (Basel)* **11**, 1288 (2021).

12. T. Wiegand, A. A. Hyman, Drops and fibers—How biomolecular condensates and cytoskeletal filaments influence each other. *Emerg. Top. Life Sci.* **4**, 247–261 (2020).

13. B. Gouveia, Y. Kim, J. W. Shaevitz, S. Petry, H. A. Stone, C. P. Brangwynne, Capillary forces generated by biomolecular condensates. *Nature* **609**, 255–264 (2022).

14. K. J. Day, G. Kago, L. Wang, J. B. Richter, C. C. Hayden, E. M. Lafer, J. C. Stachowiak, Liquid-like protein interactions catalyse assembly of endocytic vesicles. *Nat. Cell Biol.* **23**, 366–376 (2021).

15. H. Feng Yuan, B. Alimohamadi, A. N. Bakka, K. J. Trementozzi, N. L. Day, P. Fawzi, J. C. Rangamani, J. C. Stachowiak, Membrane bending by protein phase separation. *Proc. Natl. Acad. Sci. U.S.A.* **118**, e2017435118 (2021).

16. J. Agudo-Canalejo, S. W. Schultz, H. Chino, S. M. Migliano, C. Saito, I. Koyama-Honda, H. Stenmark, A. Brech, A. I. May, N. Mizushima, R. L. Knorr, Wetting regulates autophagy of phase-separated compartments and the cytosol. *Nature* **591**, 142–146 (2021).

17. Y. Fujioka, J. M. Alam, D. Noshiro, K. Mouri, T. Ando, Y. Okada, A. I. May, R. L. Knorr, K. Suzuki, Y. Ohsumi, N. N. Noda, Phase separation organizes the site of autophagosome formation. *Nature* **578**, 301–305 (2020).

18. L. Jawerth, E. Fischer-Friedrich, S. Saha, J. Wang, T. Franzmann, X. Zhang, J. Sachweh, M. Ruer, M. Ijavi, S. Saha, J. Mahamid, A. A. Hyman, F. Jülicher, Protein condensates as aging Maxwell fluids. *Science* **370**, 1317–1323 (2020).

19. A. W. Folkmann, A. Putnam, C. F. Lee, G. Seydoux, Regulation of biomolecular condensates by interfacial protein clusters. *Science* **373**, 1218–1224 (2021).

20. H. Marmor-Kollet, A. Siany, N. Kedersha, N. Knafo, N. Rivkin, Y. M. Danino, T. G. Moens, T. Olender, D. Sheban, N. Cohen, T. Dadosh, Y. Addadi, R. Ravid, C. Eitan, B. Toth Cohen, S. Hofmann, C. L. Riggs, V. M. Advani, A. Higginbottom, J. Cooper-Knock, J. H. Hanna, Y. Merbl, L. Van Den Bosch, P. Anderson, P. Ivanov, T. Geiger, E. Hornstein, Spatiotemporal proteomic analysis of stress granule disassembly using APEX reveals regulation by SUMOylation and links to ALS pathogenesis. *Mol. Cell* **80**, 876–891.e6 (2020).

21. D. W. Sanders, N. Kedersha, D. S. W. Lee, A. R. Strom, V. Drake, J. A. Riback, D. Bracha, J. M. Eeftens, A. Iwanicki, A. Wang, M.-T. Wei, G. Whitney, S. M. Lyons, P. Anderson, W. M. Jacobs, P. Ivanov, C. P. Brangwynne, Competing protein-RNA interaction networks control multiphase intracellular organization. *Cell* **181**, 306–324.e28 (2020).

22. E. I. Franses, O. A. Basaran, C.-H. Chang, Techniques to measure dynamic surface tension. *Curr. Opin. Colloid Interface Sci.* **1**, 296–303 (1996).

23. P. L. du Noüy, An interfacial tensiometer for universal use. *J. Gen. Physiol.* **7**, 625–631 (1925).

24. L. Wilhelmy, Ueber die Abhängigkeit der Capillaritäts-Constanten des Alkohols von Substanz und Gestalt des benetzten festen Körpers. *Ann. Phys.* **195**, 177–217 (1863).

25. C. M. Caragine, S. C. Haley, A. Zidovska, Surface fluctuations and coalescence of nucleolar droplets in the human cell nucleus. *Phys. Rev. Lett.* **121**, 148101 (2018).

26. C. P. Brangwynne, C. R. Eckmann, D. S. Courson, A. Rybarska, C. Hoege, J. Gharakhani, F. Jülicher, A. A. Hyman, Germline P granules are liquid droplets that localize by controlled dissolution/condensation. *Science* **324**, 1729–1732 (2009).

27. L. Zhu, T. M. Richardson, L. Wacheul, M.-T. Wei, M. Feric, G. Whitney, D. L. J. Lafontaine, C. P. Brangwynne, Controlling the material properties and rRNA processing function of the nucleolus using light. *Proc. Natl. Acad. Sci. U.S.A.* **116**, 17330–17335 (2019).

28. Simon Alberti, Amy Gladfelter, Tanja Mittag, Considerations and challenges in studying liquid-liquid phase separation and biomolecular condensates. *Cell* **176**, 419–434 (2019).

29. N. O. Taylor, M.-T. Wei, H. A. Stone, C. P. Brangwynne, Quantifying dynamics in phase-separated condensates using fluorescence recovery after photobleaching. *Biophys. J.* **117**, 1285–1300 (2019).

30. D. T. McSwiggen, M. Mir, X. Darzacq, R. Tjian, Evaluating phase separation in live cells: diagnosis, caveats, and functional consequences. *Genes Dev.* **33**, 1619–1634 (2019).

31. Florian Mueller, Davide Mazza, Timothy J. Stasevich, James G. McNally, FRAP and kinetic modeling in the analysis of nuclear protein dynamics: What do we really know? *Curr. Opin. Cell Biol.* **22**, 403–411 (2010).

32. B. S. Schuster, R. M. Regy, E. M. Dolan, A. Kanchi Ranganath, N. Jovic, S. D. Khare, Z. Shi, J. Mittal, Biomolecular condensates: Sequence determinants of phase separation, microstructural organization, enzymatic activity, and material properties. *J. Phys. Chem. B* **125**, 3441–3451 (2021).

33. P. Anderson, N. Kedersha, Stressful initiations. *J. Cell Sci.* **115**, 3227–3234 (2002).

34. P. Anderson, N. Kedersha, Stress granules: The Tao of RNA triage. *Trends Biochem. Sci.* **33**, 141–150 (2008).

35. B. Wolozin, P. Ivanov, Stress granules and neurodegeneration. *Nat. Rev. Neurosci.* **20**, 649–666 (2019).

36. D. G. A. L. Aarts, M. Schmidt, H. N. W. Lekkerkerker, Direct visual observation of thermal capillary waves. *Science* **304**, 847–850 (2004).

37. J. Pécréaux, H.-G. Döbereiner, J. Prost, J.-F. Joanny, P. Bassereau, Refined contour analysis of giant unilamellar vesicles. *Eur. Phys. J. E. Soft Matter.* **13**, 277–290 (2004).

38. N. Kedersha, M. D. Panas, C. A. Achorn, S. Lyons, S. Tisdale, T. Hickman, M. Thomas, J. Lieberman, G. M. McInerney, P. Ivanov, P. Anderson, G3BP-Caprin1-USP10 complexes mediate stress granule condensation and associate with 40S subunits. *J. Cell Biol.* **212**, 845–860 (2016).

39. W. Helfrich, Elastic properties of lipid bilayers: Theory and possible experiments. *Z. Naturforsch. C.* **28**, 693–703 (1973).

40. M. Farag, S. R. Cohen, W. M. Borcherds, A. Bremer, T. Mittag, R. V. Pappu, Condensates formed by prion-like low-complexity domains have small-world network structures and interfaces defined by expanded conformations. *Nat. Commun.* **13**, 7722 (2022).

41. A. Garaizar, J. R. Espinosa, J. A. Joseph, G. Krainer, Y. Shen, T. P. J. Knowles, R. Collepardo-Guevara, Aging can transform single-component protein condensates into multiphase architectures. *Proc. Natl. Acad. Sci. U.S.A.* **119**, e2119800119 (2022).

42. D. G. A. L. Aarts, The interface in demixed colloid–polymer systems: Wetting, waves and droplets. *Soft Matter* **3**, 19–23 (2006).

43. F. P. Buff, "The Theory of Capillarity" in *Structure of Liquids/Struktur der Flüssigkeiten*, H. S. Green, S. Ono, S. Kondo, F. P. Buff, Eds. (Springer, Berlin, Heidelberg, 1960), pp. 281–304.

44. R. Wipf, S. Jaksch, B. Stühn, Dynamics in water-AOT-n-decane microemulsions with poly(ethylene glycol) probed by dielectric spectroscopy. *Colloid Polym. Sci.* **288**, 589–601 (2010).

45. T. M. Franzmann, M. Jahnel, A. Pozniakovsky, J. Mahamid, A. S. Holehouse, E. Nüske, D. Richter, W. Baumeister, S. W. Grill, R. V. Pappu, A. A. Hyman, S. Alberti, Phase separation of a yeast prion protein promotes cellular fitness. *Science* **359**, eaao5654 (2018).

46. J. Qian, M. Hassanein, M. D. Hoeksema, B. K. Harris, Y. Zou, H. Chen, P. Lu, R. Eisenberg, J. Wang, A. Espinosa, X. Ji, F. T. Harris, S. M. J. Rahman, P. P. Massion, The RNA binding protein FXR1 is a new driver in the 3q26-29 amplicon and predicts poor prognosis in human cancers. *Proc. Natl. Acad. Sci. U.S.A.* **112**, 3469–3474 (2015).

47. T. Lu, E. Spruijt, Multiphase complex coacervate droplets. *J. Am. Chem. Soc.* **142**, 2905–2914 (2020).

48. T. Kaur, M. Raju, I. Alshareedah, R. B. Davis, D. A. Potoyan, P. R. Banerjee, Sequence-encoded and composition-dependent protein-RNA interactions control multiphasic condensate morphologies. *Nat. Commun.* **12**, 872 (2021).

49. H. Kusumaatmaja, A. I. May, R. L. Knorr, Intracellular wetting mediates contacts between liquid compartments and membrane-bound organelles. *J. Cell Biol.* **220**, e202103175 (2021).

50. T. Quail, S. Golfier, M. Elsner, K. Ishihara, V. Murugesan, R. Renger, F. Jülicher, J. Brugués, Force generation by protein–DNA co-condensation. *Nat. Phys.* **17**, 1007–1012 (2021).

51. P. Bhat, D. Honson, M. Guttman, Nuclear compartmentalization as a mechanism of quantitative control of gene expression. *Nat. Rev. Mol. Cell Biol.* **22**, 653–670 (2021).

52. A. R. Strom, A. V. Emelyanov, M. Mir, D. V. Fyodorov, X. Darzacq, G. H. Karpen, Phase separation drives heterochromatin domain formation. *Nature* **547**, 241–245 (2017).

53. T. J. Böddeker, K. A. Rosowski, D. Berchtold, L. Emmanouilidis, Y. Han, F. H. T. Allain, R. W. Style, L. Pelkmans, E. R. Dufresne, Non-specific adhesive forces between filaments and membraneless organelles. *Nat. Phys.* **18**, 571–578 (2022).

54. L.-P. Bergeron-Sandoval, S. Kumar, H. K. Heris, C. L. A. Chang, C. E. Cornell, S. L. Keller, P. François, A. G. Hendricks, A. J. Ehrlicher, R. V. Pappu, S. W. Michnick, Endocytic proteins with prion-like domains form viscoelastic condensates that enable membrane remodeling. *Proc. Natl. Acad. Sci. U.S.A.* **118**, e2113789118 (2021).

55. I. Alshareedah, M. M. Moosa, M. Pham, D. A. Potoyan, P. R. Banerjee, Programmable viscoelasticity in protein-RNA condensates with disordered sticker-spacer polypeptides. *Nat. Commun.* **12**, 6620 (2021).

56. F. M. Kelley, B. Favetta, R. M. Regy, J. Mittal, B. S. Schuster, Amphiphilic proteins coassemble into multiphasic condensates and act as biomolecular surfactants. *Proc. Natl. Acad. Sci. U.S.A.* **118**, e2109967118 (2021).

57. J. Risso-Ballester, M. Galloux, J. Cao, R. Le Goffic, F. Hontonnou, A. Jobart-Malfait, A. Desquesnes, S. M. Sake, S. Haid, M. Du, X. Zhang, H. Zhang, Z. Wang, V. Rincheval, Y. Zhang, T. Pietschmann, J.-F. Eléouët, M.-A. Rameix-Welti, R. Altmeyer, A condensate-hardening drug blocks RSV replication in vivo. *Nature* **595**, 596–599 (2021).

58. S. A. Rautu, D. Orsi, L. D. Michele, G. Rowlands, P. Cicuta, M. S. Turner, The role of optical projection in the analysis of membrane fluctuations. *Soft Matter* **13**, 3480–3483 (2017).

59. K. K. Nakashima, M. H. I. van Haren, A. A. M. André, I. Robu, E. Spruijt, Active coacervate droplets are protocells that grow and resist Ostwald ripening. *Nat. Commun.* **12**, 3819 (2021).

60. H. Turlier, D. A. Fedosov, B. Audoly, T. Auth, N. S. Gov, C. Sykes, J.-F. Joanny, G. Gompper, T. Betz, Equilibrium physics breakdown reveals the active nature of red blood cell flickering. *Nat. Phys.* **12**, 513–519 (2016).

61. R. Rodríguez-García, I. López-Montero, M. Mell, G. Egea, N. S. Gov, F. Monroy, Direct cytoskeleton forces cause membrane softening in red blood cells. *Biophys. J.* **108**, 2794–2806 (2015).

62. D. Loi, S. Mossa, L. F. Cugliandolo, Effective temperature of active matter. *Phys. Rev. E.* **77**, 051111 (2008).

63. N. Kedersha, G. Stoecklin, M. Ayodele, P. Yacono, J. Lykke-Andersen, M. J. Fritzler, D. Scheuner, R. J. Kaufman, D. E. Golan, P. Anderson, Stress granules and processing bodies are dynamically linked sites of mRNP remodeling. *J. Cell Biol.* **169**, 871–884 (2005).

64. N. Kedersha, P. Anderson, "Mammalian Stress Granules and Processing Bodies," in *Methods in Enzymology* (Academic Press, 2007), **431**, pp. 61–81; www.sciencedirect.com/science/article/pii/S0076687907310057.

65. S. A. Safran, Fluctuations of spherical microemulsions. *J. Chem. Phys.* **78**, 2073 (1983).

66. W. Häckl, U. Seifert, E. Sackmann, Effects of fully and partially solubilized amphiphiles on bilayer bending stiffness and temperature dependence of the effective tension of giant vesicles. *J. Phys. II. France* **7**, 1141–1157 (1997).

67. H. Engelhardt, H. P. Duwe, E. Sackmann, Bilayer bending elasticity measured by Fourier analysis of thermally excited surface undulations of flaccid vesicles. *J. Phys. Lett.* **46**, 395–400 (1985).

68. E. Becker, W. J. Hiller, T. A. Kowalewski, Experimental and theoretical investigation of large-amplitude oscillations of liquid droplets. *J. Fluid Mech.* **231**, 189–210 (1991).

69. E. Ziegel, Numerical recipes: The art of scientific computing. *Dent. Tech.* **29**, 501–502 (1987).
